# Does transient cART started during primary HIV infection undermine the long-term immunologic and virologic response on cART resumption?

**DOI:** 10.1186/s12879-015-0892-1

**Published:** 2015-04-10

**Authors:** Evguenia Krastinova, Remonie Seng, Jerome Lechenadec, Henri Panjo, Asma Essat, Djamila Makhloufi, Martine Obadia, Louis Bernard, Cecile Goujard, Laurence Meyer, for the ANRS PRIMO cohort

**Affiliations:** INSERM, U1018, Epidemiology of HIV and STI, CESP; University Paris-Sud, Le Kremlin-Bicêtre, France; Department of Public Health and Epidemiology, Bicêtre Hospital, AP-HP, Le Kremlin Bicêtre, France; INSERM, U1018, Gender, Sexual and Reproductive Health, Le Kremlin-Bicêtre, France; Department of Infectious and Tropical Disease, Edouard Herriot Hospital, Lyon, France; Department of Infectious and Tropical Disease, Purpan Hospital, Toulouse, France; Department of Infectious and Tropical Disease, Bretonneau Hospital, Tours, France; Department of Internal Medicine, Bicêtre Hospital, AP-HP; University Paris-Sud, Le Kremlin-Bicêtre, France

**Keywords:** Primary HIV-infection, Antiretroviral therapy, Immune response, Cohort, Long-term outcome

## Abstract

**Background:**

We explored the impact of transient cART started during the primary HIV-infection (PHI) on the long-term immunologic and virologic response on cART resumption, by comparison with treatment initiation during the chronic phase of HIV infection (CHI).

**Methods:**

We analyzed data on 1450 patients enrolled during PHI in the ANRS PRIMO cohort between 1996 and 2013. “Treatment resumption” was defined as at least 3 months of resumed treatment following interruption of at least 1 month of treatment initiated during PHI. “Treatment initiation during CHI” was defined as cART initiated ≥6 months after PHI. The virologic response to resumed treatment and to treatment initiated during CHI was analyzed with survival models. The CD4 cell count dynamics was modeled with piecewise linear mixed models.

**Results:**

136 patients who resumed cART for a median (IQR) of 32 (18–51) months were compared with 377 patients who started cART during CHI for a median of 45 (22–57) months. Most patients (97%) achieved HIV-RNA <50 cp/mL after similar times in the two groups. The CD4 cell count rose similarly in the two groups during the first 12 months. However, after 12 months, patients who started cART during CHI had a better immunological response than those who resumed cART (p = 0.01); therefore, at 36 months, the gains in √CD4 cells/mm^3^ and CD4% were significantly greater in patients who started treatment during CHI.

**Conclusion:**

These results suggest that interruption of cART started during PHI has a significant, albeit modest negative impact on CD4 cell recovery on cART resumption.

## Background

In 2013, American and French guidelines recommended universal treatment of HIV infection regardless of the CD4 cell count. Treatment initiation for patients with primary infection changed from “*should be considered optional*” to “*should be offered*” [1,2]. WHO, European AIDS clinical society and BHIVA guidelines are still based on the CD4 cell count for treatment initiation in asymptomatic patients with primary HIV infection [3,4].

Treatment initiation during primary HIV infection (PHI) has both advantages and disadvantages: it limits the loss of CD4 cells, suppresses viremia, limits the size of the latent reservoir, attenuates immune activation [5-7], and reduces infectivity [8]. Transient treatment of PHI might limit the viral setpoint and influence the CD4 cell outcome after treatment interruption, although this benefit would only be temporary [9-12]. The main disadvantage of cART initiation during PHI is the unknown risk of longer cumulative drug exposure. In addition, earlier initiation of what, for now, remains a lifelong therapy may undermine patients’ quality of life.

A small fraction of HIV-1-infected patients - so-called ‘post treatment controllers’ (PTC) - maintain viral suppression after stopping cART. This status is mostly established when treatment was initiated early during PHI [13-15]. PTC are rare and the majority of the patients will have to resume cART. There are no data on the impact of transient treatment started during PHI on long-term immunologic outcome on treatment resumption. Only one study has explored the virologic impact of cART resumption, but it did not model the CD4 cell count [12].

Enrollments in the ongoing ANRS PRIMO cohort started in 1996, providing an opportunity to analyze diverse cART modalities, including the timing of treatment initiation [16]. Here we explored the impact of transient cART started during PHI on immunovirologic responses on cART resumption, by comparison with treatment initiation during the chronic phase of HIV infection (CHI).

## Methods

### Study population

The ANRS PRIMO cohort consists of 1450 HIV-infected patients enrolled during PHI between June 1996 and December 2013 in 94 French hospitals. Primary infection is confirmed by an incomplete Western blot, or detectable p24 antigenemia, or detectable plasma viral load with a negative or weakly reactive enzyme-linked immunosorbent assay (ELISA), or an interval of less than 6 months (3 months since 2002) between a negative and positive ELISA. Clinical and biological data are collected at months 1, 3 and 6 and every 6 months thereafter, as previously described [17]. The HIV envelope gene was sequenced from frozen plasma samples collected at enrolment in the cohort and HIV tropism was determined using Geno2Pheno algorithm (FPR 10%).

All patients are antiretroviral-naïve at enrollment and give their written informed consent. The cohort was approved by the Paris-Cochin Ethics Committee. No specific recommendations for treatment initiation were given in the PRIMO cohort, apart from regularly revised, French recommendations.

### Study definitions

The date of HIV infection was estimated as the date of symptom onset minus 15 days, the date of an incomplete western blot minus 1 month, or the midpoint between a negative and a positive ELISA.

Transient cART during PHI was defined as treatment that started within 3 months after the estimated date of HIV infection, lasted at least 3 months, was interrupted for at least 1 month, and was then resumed. cART initiation during CHI was defined as initiation at least 6 months after HIV infection, for at least 3 months. cART was defined as a regimen comprising at least two nucleoside reverse transcriptase inhibitors combined with either a protease inhibitor (boosted or not) or an integrase inhibitor or a non nucleoside reverse transcriptase inhibitor (NNRTI). The CD4 cell counts and HIV loads at cART initiation/resumption are those obtained between one month before and 7 days after cART initiation.

### Statistical analyses

The response to treatment was compared between patients who started cART during CHI and those who resumed cART after transient treatment started during PHI. Time zero was the date of treatment initiation in the CHI group and the date of treatment resumption in the group with transient cART during PHI.

Baseline characteristics were compared with the Chi2 test and the Wilcoxon rank-sum test for dichotomous and continuous variables, respectively. When necessary, continuous covariates were categorized according to the median of observed values, or using published cut-off values.

Kaplan-Meier survival curves were used to analyze the time to virologic response, defined as a decrease in plasma HIV RNA to below 50 copies per milliliter, and were compared using the logrank test. Univariate and multivariate analyses were performed with Cox proportional hazards models. The proportionality assumptions were assessed by checking the log cumulative survival plots. Censoring was imposed when the patient was lost to follow-up or interrupted cART for more than 15 days. Baseline HIV load in log_10_ copies/mL was included in the model as a continuous variable after verifying the linearity assumption.

The CD4 cell count kinetics were analyzed on a square-root scale in order to obtain a normal distribution. CD4 cell gains were modeled using piecewise linear mixed-effects models in order to take into account the correlation between measurements in a given subject. The models included both fixed and random effects for the intercept and slope. The best model (Akaike’s criterion) was obtained with slope changes at M3 and M12. We modeled the CD4 cell dynamics for the first 60 months after cART initiation, during which the median number of available CD4 cell measurements was 9 per subject (IQR 6–14). Slopes of CD4 cell counts were compared between the two groups (cART initiation during CHI versus resumption after transient treatment started during PHI). Models were adjusted for age (≥40 versus <40 years), the calendar period (<2005; 2005–2007; >2007), HIV-RNA levels (≥5 log versus <5), active smoking at cART initiation/resumption, HIV transmission group (homosexual men, heterosexual men, women), geographic origin, time since HIV infection, the HIV subtype (B versus non B), and genotypic resistance at baseline.

In order to take into account the potential severity of the underlying HIV disease at baseline, the CD4 cell count at HIV primary infection diagnosis was introduced in the model. Stratified analyses were also performed, separating patients who started cART during PHI and had unfavourable baseline characteristics (CD4 < 500 cells/mm^3^ and HIV load ≥ 5 log) from their counterparts with favourable characteristics (CD4 ≥ 500 cells/mm^3^ and HIV load <5 log).

In order to study the impact of the type and duration of first-line cART started during PHI on virological and immunological outcomes after treatment resumption, we distinguished between regimens with a boosted protease inhibitor (PI) and those with a non boosted PI or NNRTI. We also distinguished, among patients who started ART during PHI, those treated for at least 24 months and those treated for less than 24 months before cART interruption. Finally, we explored the impact of the duration of cART interruption (>12 months versus ≤ 12 months).

Percentage CD4 cell counts were also modeled. The best model (Akaike’s criterion) for CD4% increase was obtained with one slope change at M3. The multivariate model comprised the same variables as in the CD4 cell count model.

The mean CD4 count and percentage evolution were depicted by plotting the mixed model predictions.

P values <0.05 were considered to denote statistically significant differences. Analyses were performed with STATA software (release 13; Stata Corp., College Station, Texas, USA).

### Sensitivity analysis

The following sensitivity analyses were conducted: ***i***) for the immune response we restricted the analyses to the subset of sustained virologic responders, i.e. patients who achieved and maintained VL <50 copies/mL throughout treatment; ***ii***) immunologic and viral analyses were repeated after excluding the 14 patients who participated in standardized treatment interruption studies (ANRS Primovac, Iliade, and ANRS Interprim [18-20]).

## Results

### Baseline characteristics

Among the 1450 patients enrolled in the ANRS PRIMO cohort, 377 started cART during CHI, while 136 patients started a second course of ART after transient treatment started during PHI. The two populations differed in terms of their baseline characteristics, as expected. Patients who initiated transient cART during PHI had lower CD4 cell counts and higher viral loads at cohort entry than those who started ART during CHI (Table 1). No difference was found according to age, baseline genotypic resistance, HIV subtype (B versus non B) and HIV-tropism (CCR5-tropic versus CXCR4 or dual X4/DM-tropic virus). Transient cART started during PHI began a median of 1.3 months (IQR 1.1-1.7) after HIV infection and lasted a median of 20 months (14–37). The median duration of ART interruption was 19 months (4–43 months). The median year of cART initiation during PHI was 2001 (1998–2003).

**Table 1:** **Baseline cohort characteristics according to cART initiation: cART resumption after transient treatment started during PHI versus cART initiation during CHI**

	**cART resumption after transient ART during PHI**	**cART initiation during CHI**	**p value**
	**N = 136**	**N = 377**	
**Sex, % (n)**			
Male	80 (107)	85 (320)	0.2
**Age at enrolment**			
Median (IQR), years	37 (31–44)	36 (30–43)	0.2
Transmission group, % (n)**			0.2
Homosexual male	64 (79)	74 (268)	
Heterosexual male	14 (17)	11 (39)	
Female	22 (28)	15 (56)	
**Place of birth, % (n)**			0.06
France	80 (107)	87 (327)	
Sub-Saharan Africa & other	20 (26)	13 (48)	
**Education, % (n)**			0.06
Primary	11 (14)	9 (32)	
Secondary	34 (44)	46 (171)	
University	55 (72)	45 (169)	
**Presence of baseline genotypic resistance % (n)**	13 (10)	14 (50)	0.2
**HIV subtype B, % n**	79 (104)	77 (280)	0.5
HIV tropism, % (n)*			0.5
CCR5-tropic	89 (64)	86 (274)	
CXCR4-tropic or dual X4/DM	11 (8)	14 (43)	
**Hepatitis C co-infection, % (n)**	1.4 (5)	0.8 (1)	0.6
**Active smoking**	40 (51)	43 (157)	0.5
**CD4 cell count at PHI diagnosis**			
Median (IQR) cells/mm3	473 (323–597)	544 (420–697)	<0.001
**HIV load at PHI diagnosis**			
Median (IQR) log_10_c/mL	5.4 (4.9-5.9)	4.9 (4.2-5.4)	<0.001

*Data on HIV tropism was available for 389 patients (338 CCR5-tropic and 51 CXCR4-tropic).

**In 26 patients the transmission mode was other (transfusion etc.) or missing.

Table 2 compares the characteristics at cART resumption of patients who received transient cART started during PHI and the baseline characteristics of patients who started treatment during CHI. The respective median CD4 cell counts were 303/mm^3^ (246–442) and 332/mm^3^ (248–426), p = 0.90, and the respective median viral loads were 4.7 log_10_ (4.2-5.3) and 4.9 log_10_ copies/mL (4.4-5.3) (p = 0.7).

**Table 2:** **Patient characteristics at cART resumption after transient cART started during PHI versus cART initiation during CHI**

**Characteristics** ^**a**^	**ART resumption after transient cART during PHI, N = 136**		**cART initiation during CHI, N = 377**		**p value**
**Age, years**	43	(37–50)	40	(34–47)	<0.0001
**CD4 cells/mm3** ^**b**^	303	(246–442)	332	(248–426)	0.9
**HIV load log**_**10**_ **copies/mL**	4.7	(4.2-5.3)	4.9	(4.4-5.3)	0.7
**Time between HIV infection and cART resumption/initiation, months**	46	(26–72)	23	(12–42)	<0.0001
**Year of cART resumption/initiation**	2006	(2003–2010)	2009	(2007–2010)	<0.0001
**Duration of cART after resumption/initiation, months**	47	(23–66)	32	(18–51)	0.0002
**Duration of viral control on cART, months**	45	(22–56)	30	(18–49)	0.0007
**cART regimen % (n)**					
Boosted protease inhibitor (PI) or anti- integrase	43	(58)	59	(223)	<0.0001

^a^Data are medians (IQR) or % (n).

^b^Baseline CD4 cell count and HIV load values were obtained between 1 month before cART initiation and 7 days following cART initiation; the number of corresponding CD4 cell counts was N = 77 for patients treated during PHI and N = 234 for patients treated during CHI.

The median time since PHI was 46 months (26–72) at cART resumption and 23 months (12–42) at cART initiation during CHI. Patients who resumed cART were older than those who initiated cART during CHI while they were the same age at PHI diagnosis. They received boosted PIs and integrase inhibitors less frequently (43% vs 59%, p < 0.0001), probably owing to differences in the calendar periods (the median year of cART resumption/initiation was 2006 and 2009, respectively). Resumed treatment lasted significantly longer than treatment initiated during CHI: median (IQR) 47 months (23–66) versus 32 months (18–51) (p = 0.0002).

### Virologic response

**Figure 1:**
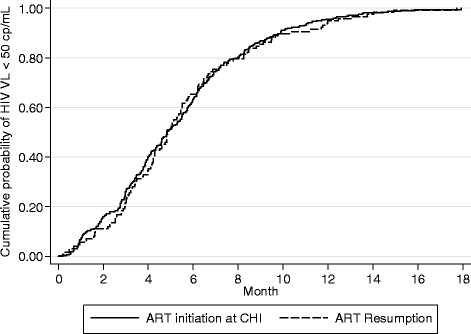
Kaplan-Meier functions of time to HIV RNA < 50 copies/mL according to the timing of cART (resumption after transient treatment during PHI versus initiation during CHI), log rank test, p = 0.5.

At 12 months of cART, VL was < 50 copies/mL in 130 (97%) patients who resumed cART and in 369 (97%) patients who started cART during CHI. The median (IQR) time taken to reach <50 copies/mL was respectively 5.0 months (3.2-7.6) and 5.0 months (3.0-7.4), logrank test, p = 0.6. (Figure 1); the crude hazard ratio (HR) was 0.9 (95% CI 0.8-1.1). Similar results were obtained after adjusting for age (≥40 versus <40 years), HIV load (log_10_copies/mL) and the CD4 cell count at cART initiation, the period of cART initiation (<2005; 2005–2007; >2007), the HIV transmission group, place of birth, education, time since HIV infection, the HIV subtype (B versus non B) and baseline genotypic resistance. Adjustment for the type of cART regimen instead of the calendar period led to similar conclusions. As expected, higher viremia at cART initiation was predictive of slower viral suppression (HR = 0.8 for a one-log increase in HIV RNA load (95% CI 0.7-0.9), p = 0.001). Baseline genotypic resistance was associated in multivariate analysis with slower viral suppression (HR 0.7 (95% CI 0.5-0.9), p = 0.02). No other parameter included in the multivariate analysis was associated with the time to viral suppression.

Considering the type of cART regimen and the duration of transient cART started during PHI did not modify the conclusion.

### Immunologic response

**Figure 2:**
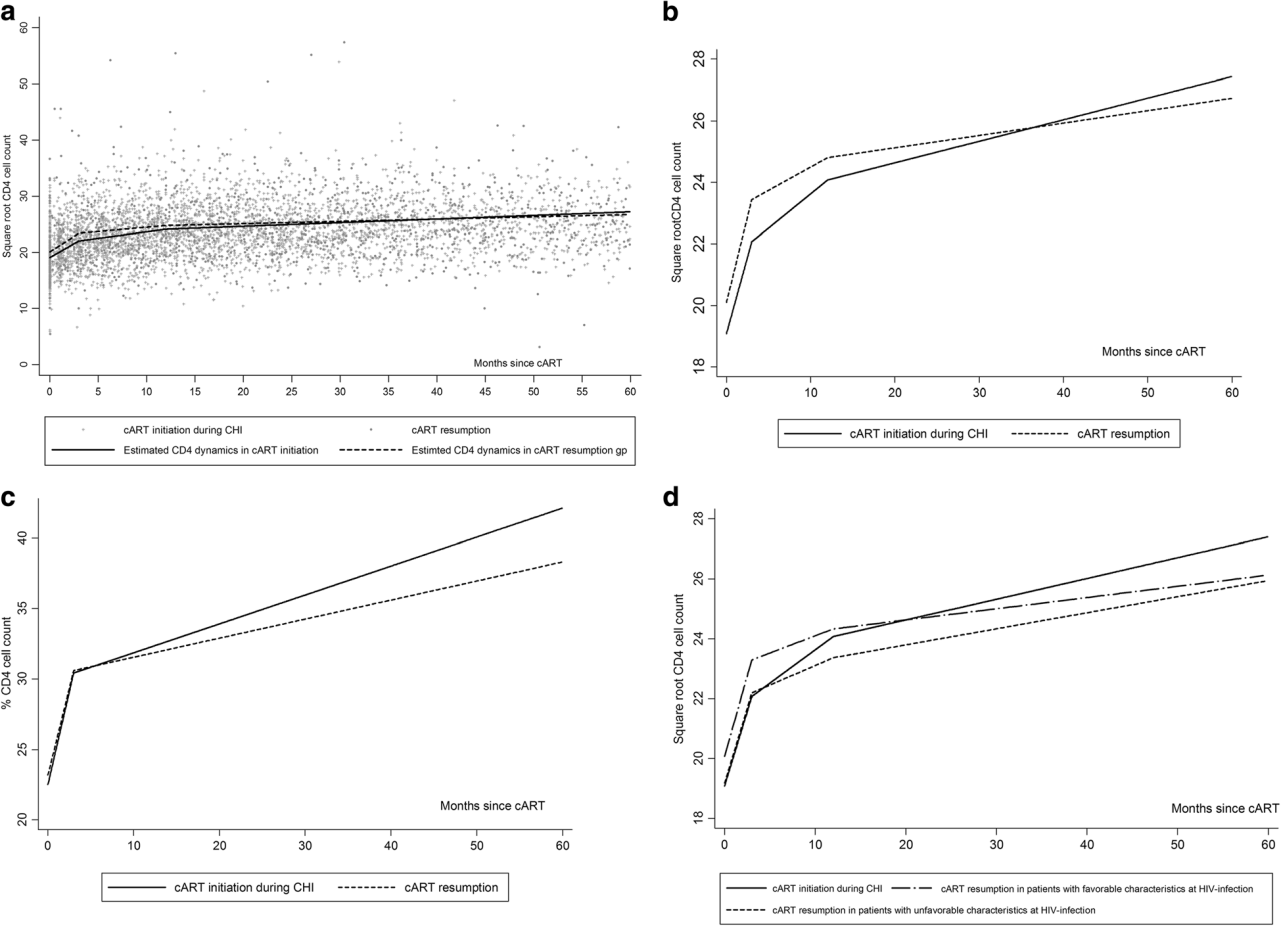
Estimated CD4 count evolution on cART (in square root scale) from the piecewise linear mixed-effects model according to the mode of cART initiation. **a**/ cART resumption after transient treatment started during PHI versus cART initiated during CHI and the observed CD4 count values; **b**/ Estimated CD4 count evolution on cART (in square root scale) from the piecewise linear mixed-effects model according to mode of cART initiation: cART resumption after transient treatment started during PHI versus cART initiated during CHI; **c**/ Estimated CD4 count percentage evolution on cART from the piecewise linear mixed-effects model according to the mode of cART initiation: cART resumption after transient treatment started during PHI versus cART initiated during CHI; **d**/ Estimated CD4 cell count evolution in patients who started cART during PHI with unfavourable ((cutoffs CD4 < 500 cells/mm^3^ and HIV load ≥ 5 log) versus favorable baseline characteristics (cutoffs CD4 ≥ 500 cells/mm^3^ and HIV load < 5 log) versus patients who started cART during CHI.

The modelled CD4 dynamics after cART initiation are shown in Figure 2. Trends in the CD4 cell count were analyzed with a piecewise linear mixed-effects model with 3 slopes (0–3 months, 3–12 months, and >12 months). The first two slopes did not differ significantly between cART resumption and cART initiation during CHI, whereas the third slope did: after 12 months, patients who initiated cART during CHI had a better immunological response (+0.070 √CD4count/month) than those who resumed cART (+0.041√CD4count/month), p = 0.01 (Table 3). Therefore, at 12 months of cART the CD4 cell count gain did not differ significantly between cART resumption and cART initiation during CHI while, at 36 months of cART the CD4 cell count gain was significantly higher when cART was started during CHI, with a difference of 1.202 √CD4count (standard error (SE) = 0.50), p = 0.02, and 1.165 √CD4count (SE = 0.52), p = 0.03 in univariate and multivariate analyses, respectively. For example, the model predicted that a patient who started cART during CHI at 300 CD4/mm3 would reach 431, 517 and 625 CD4/mm3 at 3, 12 and 36 months, respectively, while a patient who resumed cART at 300 CD4/mm3 after transient treatment started during PHI would reach 441, 517 and 572 CD4/mm3.

**Table 3:** **Immunologic response to cART according to mode of cART initiation (cART initiation during CHI versus cART resumption) from linear mixed-effects models with 3 slopes**

**Parameter**	**Univariate estimate**	**SE**	***P*** **value**	**Adjusted estimate** ^**b**^	**SE**	***P*** **value**
**Intercept √CD4 cART (reference)**	19.085	0.235	<0.0001			
cART resumption vs Initiation during CHI^a^	1.012	0.482	0.04	−0.067	0.347	0.8
**Slope 1 (0-3mo) √CD4/month (ref)**	0.995	0.058	<0.0001			
cART resumption vs initiation during CHI^a^	0.118	0.126	0.4	0.063	0.129	0.5
**Slope 2 (3–12 mo) √CD4/month (ref)**	0.222	0.018	<0.0001			
cART resumption vs initiation during CHI^a^	−0.071	0.036	0.06	−0.056	0.038	0.1
**Slope 3 > 12 mo √CD4/month (ref.)**	0.070	0.007	<0.0001			
cART resumption vs initiation during CHI^a^	−0.029	0.011	0.01	−0.024	0.012	0.04

^a^Difference between cART resumption and cART initiation during CHI (reference).

^b^Adjusted in multivariate analysis, for age (≥40 versus <40 years), calendar period, HIV-RNA level (≥5 log versus <5), active smoking at cART initiation/resumption, HIV transmission group (homosexual men, heterosexual men, women), geographic origin, time since HIV infection, HIV subtype (B versus non B), baseline genotypic resistance, and CD4 cell count at PHI diagnosis.

No marked change in these results was found after adjusting for age, the calendar period, HIV-RNA level, active smoking at cART initiation/resumption, the HIV transmission group, geographic origin, time since HIV infection, HIV subtype, baseline genotypic resistance, and the CD4 cell count at PHI.

When the analyses were repeated for the subset of patients with sustained viral responses (HIV-RNA < 50 copies/mL), we still found a better long-term immunological response in patients who started cART during CHI than in those who resumed cART.

CD4% was modelled with 2 slopes (0–3 months and >3 months) (Figure 2c). No difference between the two groups was observed in the first slope (up to 3 months), while after 3 months the slope was 0.24%CD4/month after cART initiation during CHI and 0.16%CD4/month after cART resumption, p = 0.001. Adjustment for the same variables as in the CD4 cell count analyses did not affect the results. The difference in the CD4% gain at 36 months was 2.97 higher after cART started during CHI than after cART resumption, p < 0.0001.

We still found better immune reconstitution in patients who started cART during CHI after stratifying for unfavourable versus favorable baseline characteristics (Figure 2d) and after taking into account the duration of cART interruption (>12 months versus ≤ 12 months) and the duration of transient cART during PHI (>24 months versus ≤ 24 months).

## Discussion

The virologic response to cART, analyzed in terms of the time taken to achieve HIV RNA <50 copies/mL, was similar in patients who resumed cART after transient treatment started during the primary phase of HIV infection and those who started cART during the chronic phase. The short-term immune response was also similar, being characterized by a rapid gain in CD4 cells during the first 3 months. In contrast, subsequent gains in the CD4 cell count and percentage were larger in the patients who started cART during CHI. These differences persisted after taking into account the patients’ characteristics at PHI (CD4 cell count and viral load) as a proxy for the severity of their underlying HIV disease.

This study is the first to suggest that interruption of treatment started during PHI may have a detrimental impact on the long-term immune response on treatment resumption. Randomised therapeutic trials conducted during PHI usually focus on the degree of viral rebound and CD4 cell loss after cART interruption, and not on long-term trends in CD4 cell numbers after treatment resumption. The remarkably lengthy follow-up of our cohort offers a possibility to explore this issue. We also studied the CD4 cell percentage dynamics, which is rarely reported despite being informative of immune reconstitution and complementary to the CD4 cell count [21].

The CD4 cell dynamics observed here are concordant with published data on treatment initiation during chronic HIV infection [22-26]. The overall immune response to treatment varies according to the CD4 cell count at cART initiation [22,25-27]. Patients with an initial count of 250–500 CD4 cells/mm^3^ will gain about 200 cells/mm^3^ during the first year of cART [27]. Reconstitution of the CD4 cell pool exhibits a biphasic pattern, with a rapid increase during the first 3 months, due to redistribution of memory cells from lymphoid tissue, followed by a substantially slower increase [22,28]. In the CASCADE collaboration, the slope of CD4 cell recovery during treatment started in CHI was similar to that observed here, with +0.95 √CD4count/month in the first 3 months, and +0.105√CD4count/month thereafter [23].

It is widely agreed that interruption of long-term cART started during CHI is harmful. In the CASCADE collaboration and SMART trial, poorer immune reconstitution was observed after ART resumption than during first-line cART initiated during CHI [23,29]: two years after cART resumption the CD4 cell count had returned to the pre-interruption level in fewer than half of the patients [30].

The difference in CD4 cell recovery observed here between patients who resumed cART after transient treatment started during PHI and those who started cART during CHI might have resulted indirectly from drug resistance following cART interruption, yet no difference in the viral response was observed. Moreover, in the sensitivity analysis restricted to sustained viral responders, the CD4 count slope was still shallower in the cART resumption group.

The detrimental consequences of discontinuing cART started during CHI have been explained in terms of increases in markers of inflammation, coagulation and immune activation, coinciding with the viral rebound after cART interruption [31,32]. A similar inflammation/activation phenomenon might also explain the results obtained here after interruption of transient cART initiated during PHI. We could also argue whether patients who initiated ART during primary HIV infection might have had a more “pro-inflammatory phenotype”, which caused lower long-term CD4 increases though the effect of pro-inflammatory cytokines, such as TNFα. There is evidence that TNFα might affect CD4 count after cART interruptions in CHI [32]. The levels of T cell activation and inflammatory cytokines (IL-1α, eotaxin and IL-7) during PHI have been found to be strong independent predictors of the rate of spontaneous CD4 cell count decline in untreated patients, but there are no such data for patients who interrupt transient cART initiated during PHI [32-34]. In the ANRS Interprim trial, in which serial short interruptions were programmed after treatment initiation during PHI, a gradual decrease in the mean CD4 cell count and percentage was observed over time, with no return to the mean values reached before the first interruption [20].

One limitation of our work, as in any observational study, is that the patients who interrupted cART after transient treatment started during PHI might have had different prognostic status from those who started cART during CHI. However, complementary analyses that took into account potentially unfavourable characteristics at HIV infection, as well as the duration and type of first-line cART regimen and the duration of cART interruption, yielded the same conclusions. It might also be argued that the patients who started treatment during CHI could have differed from their “usual” counterparts as they had been followed in the cohort since PHI. However, the viral response to cART initiated during CHI was similar to that observed in randomised trials [35], and the immune response was compatible with published data [22-24,28]. Of note, the CD4 cell counts were very similar in the patients who resumed cART and those who started cART during CHI, at around 300 cells/mm^3^, a value in line with the evolving thresholds at which French guidelines recommended treatment initiation during the 1996–2013 study period.

It may be argued that the difference in immune reconstitution is not clinically pertinent: indeed, after 36 months the mean CD4 cell count was above 500 cells/mm^3^ in both groups. The difference between the groups was about 53 CD4 cells/mm^3^ after 36 months of cART for a patient starting at 300 cells/mm^3^. However, recent data suggest that complete immune reconstitution might be defined by higher levels than the commonly accepted count of 500 cells/mm^3^ [36], which would make our results even more relevant.

The possibility of inducing long-term immuno-virologic control by initiating ART at an early stage of HIV infection is exciting, and further research is needed to identify future post-treatment controllers (PTC) and those achieving recently described functional cure [37].

## Conclusion

Our results show that cART interruption after transient cART started during PHI has a significant, albeit modest negative impact on CD4 count reconstitution after cART resumption, an encouraging finding in the context of therapeutic trials attempting to induce PTC status. Pending the results of these studies, our findings confirm that, once initiated, cART should not be interrupted, except in research settings and under close medical surveillance.
